# Chronic Remote Ischemic Conditioning Is Cerebroprotective and Induces Vascular Remodeling in a VCID Model

**DOI:** 10.1007/s12975-017-0555-1

**Published:** 2017-07-28

**Authors:** Mohammad Badruzzaman Khan, Sherif Hafez, Md. Nasrul Hoda, Babak Baban, Jesse Wagner, Mohamed E. Awad, Hasith Sangabathula, Stephen Haigh, Mohammed Elsalanty, Jennifer L. Waller, David C. Hess

**Affiliations:** 1 Departments of Neurology, Medical College of Georgia, Augusta University, 1120 15th Street, CA1053, Augusta, GA 30912 USA; 2 Department of Medical Laboratory, Imagine and Radiologic Sciences, Augusta University, Augusta, GA 30912 USA; 3 Department of Oral Biology, Augusta University, Augusta, GA 30912 USA; 4 Vascular Biology Center, Augusta University, Augusta, GA 30912 USA; 5 Departments of Biostatistics, Augusta University, Augusta, GA 30912 USA

**Keywords:** Chronic remote ischemic conditioning (C-RIC), Cerebral blood flow (CBF), Vascular contributions to cognitive impairment and dementia (VCID), Angiogenesis, collateral remodeling, white matter degeneration

## Abstract

Vascular contributions to cognitive impairment and dementia (VCID) make up 50% of the cases of dementia. The purpose of this study was to determine the effect of chronic remote ischemic conditioning (C-RIC) on improving long-term (6 months) outcomes and cerebral blood flow (CBF) and collateral formation in a mouse model of VCID. Adult C57BL/6J male mice (10 weeks) were randomly assigned to four different groups: (1) sham-bilateral carotid artery stenosis (BCAS), (2) BCAS + sham RIC, (3) BCAS+C-RIC for 1 month (1MO), and (4) BCAS+C-RIC-4 months (4MO). CBF, cognitive impairment, and functional outcomes were performed up for 6 months after BCAS surgery. The expression of CD31, α-SMA, and myelin basic protein (MBP) was assessed by immunohistochemistry (IHC). Additional set of mice were randomized to sham, BCAS, and BCAS+C-RIC. The cerebrovascular angioarchitecture was studied with micro-CT. RIC therapy for either 1 or 4 months significantly improved CBF, new collateral formation, functional and cognitive outcomes, and prevented white matter damage. There was no difference between C-RIC for 1 or 4 months; IHC studies at 6 months showed an increase in brain CD31 and α-SMA expression indicating increased angiogenesis and MBP indicating preservation of white matter in animals receiving RIC. One month of daily RIC is as effective as 4 months of daily RIC in improving CBF, angiogenesis, and long-term functional outcomes (6 months) in a VCID model. This suggests that 1 month of RIC is sufficient to reduce cognitive impairment and induce beneficial cerebrovascular remodeling.

**Electronic supplementary material:**

The online version of this article (doi:10.1007/s12975-017-0555-1) contains supplementary material, which is available to authorized users.

## Introduction

The prevalence of dementia is expected to triple by 2050, a major threat to the world’s public health. Vascular dementia makes up to 20% of the cases of dementia, and mixed dementia (vascular and Alzheimer’s) is estimated to be up to 50% of the cases of dementia [1]. Moreover, cerebral ischemia worsens Alzheimer’s disease (AD) and triggers its clinical expression [2]. Vascular contributions to cognitive impairment and dementia (VCID) is a term that encompasses the vascular factors and vascular pathology that underlie the clinical spectrum from mild cognitive impairment to dementia. VCID is a major research focus of the National Institute of Neurological Diseases and Stroke [3, 4]. Other than controlling the comorbid vascular risk factors, there is no known effective treatment for VCID. However, clinical observational studies strongly suggest *that physical exercise is effective* at reducing progression of cognitive decline and dementia [5].

Reduction of cerebral blood flow (CBF) may be the key precipitating event in AD and VCID [1]. Hypoperfusion appears to be an early finding that plays a pathophysiological role in the development of white matter (WM) damage [6]. Low blood flow by MRI perfusion or MRI arterial spin labeling (ASL) is predictive of white matter lesions [7, 8]. A penumbra exists around white matter lesions that expand in relation to low CBF [8]. An animal model for VCID is essential to test new interventions.

In a recent review of all mouse models for VCID, Bink et al. determined the mouse bilateral carotid artery stenosis (BCAS) model to be the most valid [9]. This model reproduces the WM damage, cerebral hypoperfusion, inflammation, BBB damage, and cognitive deficits of the human condition [10, 11]. There are also fibrinoid changes in the small vessels of the brain with gliosis and disruption of aquaporin polarization [12]. With these small vessel changes, the BCAS model may be useful to test interventions to treat small vessel disease of the brain [12].

Remote limb ischemic conditioning (RIC) is the simple, inexpensive, and safe use of repetitive inflation of a blood pressure (BP) cuff on the arm or leg to protect distant organs such as the brain from ischemic injury. Chronic RIC (C-RIC) is the repetitive use of daily RIC for periods of weeks or months. RIC shares common mechanisms with physical exercise and may be viewed as an “exercise equivalent” [13, 14]. We previously showed in a BCAS mouse model that C-RIC for 2 weeks increased CBF in a sustained fashion, reduced WM damage, improved cognitive performance, and reduced accumulation of amyloid-beta 42 protein (Aβ_42_) in the brain [15].

However, whether RIC can induce long-term neuroprotection and how long RIC would need to be applied are not known. This study aimed to determine if C-RIC can induce long-term (at 6 months) neurovascular protection, and if 1 month of daily RIC is as effective as 4 months in a BCAS model. Our secondary aim was to determine whether C-RIC-induced neuroprotection is attributed to RIC-induced vascular remodeling and angiogenesis in the brain, similar to what is seen with chronic physical exercise.

## Materials and Methods

### Animal Models and Experimental Groups

The animals were housed at Augusta University’s animal care facility, which is approved by the American Association for Accreditation of Laboratory Animal Care. This study was conducted in accordance with the National Institute of Health guidelines for the care and use of animals in research, and all protocols were approved by the institutional animal care and use committee. All the STAIR (Stroke Therapy Academic Industry Roundtable) and RIGOR recommendations and guidelines regarding randomization, blinding, and statistical analysis were followed in this study [16, 17].

### Determine the Effect of Chronic RIC on Cognitive Impairment, Functional Outcomes, and CBF

Forty male mice were randomized into four groups: (1) sham (operated group for procedures of BCAS), (2) BCAS and sham RIC, (3) BCAS treated with daily RIC for 1 month post BCAS surgery (BCAS + RIC-1MO, *N* = 10), and (4) BCAS treated with daily RIC for 4 months post BCAS surgery (BCAS + RIC-4MO, *N* = 10). The outcomes were assessed by a blinded investigator. Cognitive function, functional outcomes, and CBF changes were considered as the primary outcomes and were determined at 4 and 6 months after BCAS, with either 1MO or 4MO RIC. Thereafter, the animals were sacrificed, the blood was collected for plasma nitrite estimation, and the brains were isolated and dissected for IHC (Schematic representation of the study plan in Supplemental Fig. 1).

### Bilateral Carotid Artery Stenosis Surgical Procedure

BCAS was performed as previously described [10, 15]. In brief, animals were anesthetized with 2% isofluorane and both common carotid arteries (CCAs) were exposed by a midline cervical incision. Customized microcoils, specially designed to mimic a VCID model in the mice which was made of steel wire with an inner diameter of 0.18 mm, was twined by rotating around both right and left CCA at 30 min interval.

### Non-Invasive RIC Therapeutic Methods

Non-invasive RIC therapy method was performed as published elsewhere [18, 19] with a programmable cuff or its sham procedure with a cuff that did not inflate or deflate (*see detail in “* *Supplemental Methods* *”*) [15]. Bilateral RIC therapy was performed in both hind limbs simultaneously (4 cycles × 5 min duration of each cycle inflate and deflate × 5 min interval between each cycle) daily for 1 month (BCAS+RIC-1MO) or 4 months (BCAS+RIC-4MO) after 1 week from BCAS surgery.

### Cerebral Blood Flow by Laser Speckle Contrast Imager

Cerebral blood flow (CBF) was measured using high-resolution Laser Speckle Contrast Imager (LSCI) (PSI system, Perimed Inc.) at different time points as indicated in the figure and as previously reported by us [15]. Mice were placed on a warming pad and thermostatically controlled at around 37 °C to avoid the effect of body temperature during the measurement of CBF.

### Estimation of Nitrite in Plasma

Plasma NO_*x*_ (NO_2_^−^ + NO_3_^−^) levels were measured by NO-specific chemiluminescence, as described previously [20] with slight modification. Briefly, 100 ul of plasma were mixed with twice volumes of ethanol (100%) and kept at −20 C for 40–60 min followed by centrifuged at 13000 rpm for 10 min to remove protein as pellet. The supernatant (100 ul) was taken and injected to measure nitrite. The nitrite levels were measured by NO Analyzer 280i (GE Analytical Instruments, CO, USA). The level of nitrite was expressed in nanomolar.

### Behavioral Test: Functional Outcomes and Cognitive Test

An investigator who was blinded to the experimental design and behavioral test or treatment including NOR test, [21] Y-maze test, [22] beam walk, [23] and wire hanging test [24] (Detailed procedures are explained in the “Supplemental Methods.”). All groups were examined at 4 and 6 months after sham surgery, BCAS surgery, and with RIC (1MO or 4MO). NOR and Y-maze test were performed for cognitive test where beam walk test required mice to balance on a wooden beam to evaluate any gait abnormality and wire hanging test for muscular or motor function.

### Histological and Immunohistochemical Assessment

Immunohistochemistry was performed according to the protocol previously described [15] with slight modification. Both paraffin and cryo-sections were used for immunohistochemistry and immunofluorescence with similar anatomic features (see the “Supplemental Methods” for details). The primary antibodies were anti-myelin basic protein (MBP) (C-16 clone, SC-13914, Santa Cruz, CA, USA; 1:100 dilution); rat anti-platelet endothelial cell adhesion molecule [PECAME-1 (CD31), BD no. 550274, USA,1:200 dilution], with mouse monoclonal anti-α-smooth muscle actin [α-SMA, SC-53142, Santa Cruz, CA, USA; 1:50 dilution]; and anti-platelet-derived growth factor [PDGFR-β (958): SC-432, Santa Cruz, CA, USA; 1:200 dilution], isolectin B4 conjugates (Invitrogen, molecular probe Life Technology, IB4 no. 121411). For biotinylated immunostaining, the brain sections were incubated in the anti-MBP primary antibodies and using the avidin–biotin–peroxidase complex method with diaminobenzidine (DAB) as the chromogen. The immunostaining was carried out using the ABC kit system (Vector, Burlingame, CA, USA). After staining, the sections were counterstained with Harris hematoxylin (cat. no. HHS35-1L; Sigma) for few seconds. The sections were then dehydrated rapidly through ethanol and xylene and mounted with VectaMount medium (Vector).

### Assessment of New Collateral Formation and Angiogenesis: Micro-CT and BriteVu Methods

Twenty-seven male mice were randomized into three groups: (1) sham, (2) BCAS and sham RIC (BCAS), and (3) BCAS treated with daily RIC for 3 weeks post BCAS surgery (BCAS+RIC). Three weeks after BCAS, blood was collected either from the eye (retro orbital) for flow cytometry analysis of endothelial progenitor cells (EPCs) and macrophages, or from the heart plasma nitrite estimation. Animals were transcardially perfused with heparinized saline to flush out the blood, followed immediately by freshly prepared BriteVu™ solution (Scarlet Imaging, LCC; Murray, UT, USA) according to manufacturer instruction. Carcasses were kept on ice at 4 °C overnight to ensure solidification of BriteVu dye prior to imaging. After 24 h, brains were isolated, fixed with 4% PFA for 48 h, and then saved in 70% alcohol till the time of imaging. SkyScan 1174 (Bruker micro-CT/formerly known as SkyScan, USA) is used for imaging. SkyScan is associated with a full-range software that provides fast volumetric reconstruction of 2D/3D quantitative analysis and realistic 3D visualization of cerebrovasculature. Outcomes: (1) full 3D picture of cerebrovasculature and (2) quantification of vascular number, vascular volume, vascular density, lumen diameter, and formation of new collaterals.

### Evaluation of EPCs and Macrophages

Whole blood (WB) was collected using heparinated microtubes as described previously with slight modification [25, 26]. One hundred fifty milliliters of WB were then incubated with antibodies for EPC markers including CD31, CD34, VEGFR2, and surface markers for M1/M2 macrophages, CD11b, F4/80, and CD206 (eBioscience, USA) for 20 min on ice in the dark. After washing was completed, cells were fixed and permeabilized using fix/perm concentrate (eBioscience, USA) before incubation with antibodies for intracellular staining of TNFα (for M1 macrophages) and IL10 (for M2 macrophages). Samples were then washed and run through a four-color flow cytometer (FACSCalibur, BD Biosciences), and data were collected using the CellQuest software. Samples were double-stained with control IgG and cell markers to assess any spillover signal of fluorochromes. Proper compensation was set to ensure that the median fluorescence intensities of negative and positive cells were identical and then was used to gate the population. Gating excluded dead cells and debris using forward and side scatter plots. To confirm the specificity of primary antibody binding and rule out non-specific Fc receptor binding to cells or other cellular protein interactions, negative control experiments were conducted using isotype controls matched to each primary antibody’s host species, isotype, and conjugation format.

### Statistical Analysis

All statistical analysis was performed using SAS 9.4, and statistical significance was assessed using a significance level of 0.05. To examine differences between the groups (sham, BCAS, BCAS+RIC-1MO, BCAS+RIC-4MO) for nitrites, a one-way ANOVA was used. If the overall test for the one-way ANOVA was statistically significant a Tukey–Kramer multiple comparison procedure was used to examine differences between the four groups. For cerebral blood flow, beam walk, hanging wire, NOR, and Y-maze measures, a repeated measures mixed model was used to examine differences between the four groups over time (CBF measurement times: baseline, post surgery, 4 months, 6 months; all other outcomes measurement times: 4 months, 6 months). Main effects of group and time as well as the two-factor interaction between group and time were included in the model. For CBF, an auto-regressive order 1 correlation structure fit the data best and was used to account for the correlation between measurement times. For the beam walk, hanging wire, NOR discrimination index, and Y-maze measures, an unstructured correlation structure was used as there were only two measurement times. Of statistical interest was the F-test for the two-factor interaction between group and time, and if it is statistically significant, it indicates that the change in the outcome over time is different for the four groups. To examine differences between groups within measurement time and between measurement times within group, a Bonferroni adjustment to the overall alpha level was used to control for the multiple post hoc pair-wise tests as not all pair-wise tests were of interest. For CBF, the Bonferroni adjusted alpha is 0.0010 and for the other outcomes, the Bonferroni adjusted alpha is 0.0031.

## Results

### RIC Improved Cerebral Blood Flow

**Fig. 1:**
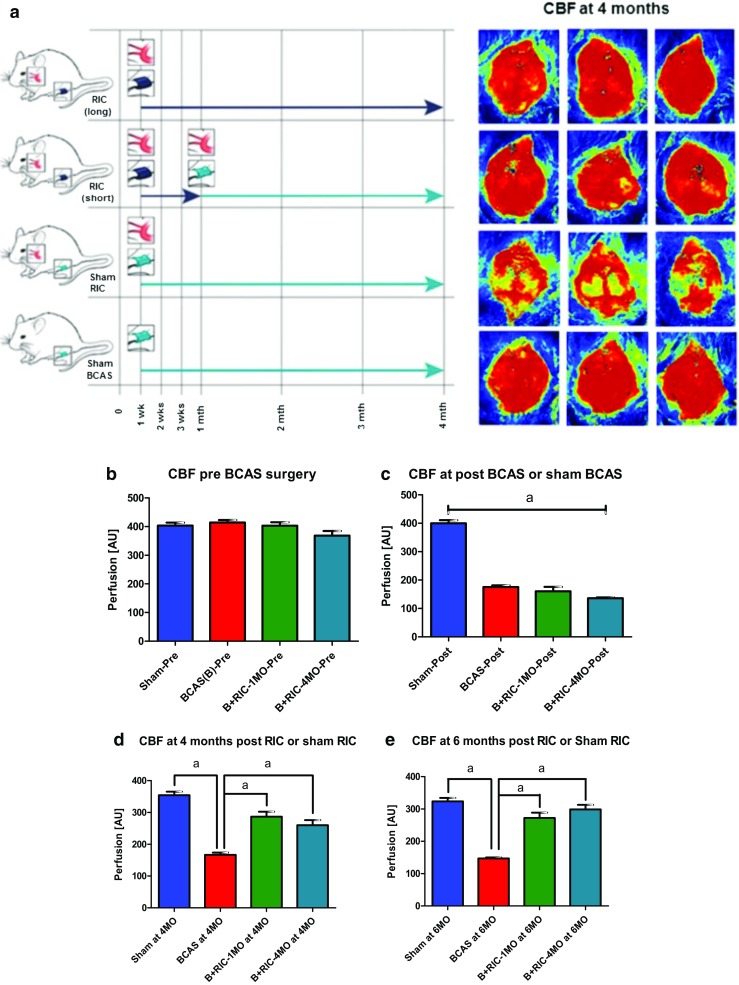
Measuring of CBF changes by Laser Speckle Contrast Imager (LSCI), remote ischemic conditioning (RIC) (1-MO and 4-MO therapy at 4MO and 6MO) increases cerebral blood flow (CBF) in bilateral carotid artery stenosis (BCAS) mice. **a** Mice underwent BCAS and were randomized to RIC daily for 4 months (long with *dark blue arrow*, *top row*), RIC daily for 1 month (short with *dark blue arrow*, *second row*), or sham RIC (*green arrow*, *third row*). The *bottom row* shows mice with sham BCAS surgery (no coils). CBF was measured at 4 and 6 months (Supplemental Fig. 2) in all mice. Daily RIC for 1 month produced similar increases in CBF to daily RIC for 4 months. *Red* indicates higher blood flow. **b**–**e** Absolute value of cerebral perfusion in perfusion unit (PU) at different time points where pre (prior to BCAS), post (after BCAS and prior to RIC), at 4 months (4MO) and at 6 months (6MO); pre and post sham operation, BCAS with sham RIC; and BCAS+RIC with 1MO and 4MO. Using repeated measures mixed models, there is no significant difference between CBF in the 1MO and 4MO therapy groups but both groups are significantly higher than the BCAS sham group at 4MO and at 6MO (*N* = 7 to 10/groups; ^a^ *p* < 0.0001 vs BCAS+sham-RIC; ^a^ *p* < 0.0001 vs BCAS+RIC-1MO and BCAS+RIC-4MO)

C-RIC therapy after BCAS increased CBF compared to sham RIC (Fig. 1a–e; Supplemental Fig. 2). CBF was significantly increased in both 1MO and 4MO RIC therapy as compared to BCAS-sham RIC. Moreover, 1MO of RIC was as effective as 4MO RIC in improving CBF at 6 months.

**Fig. 2:**
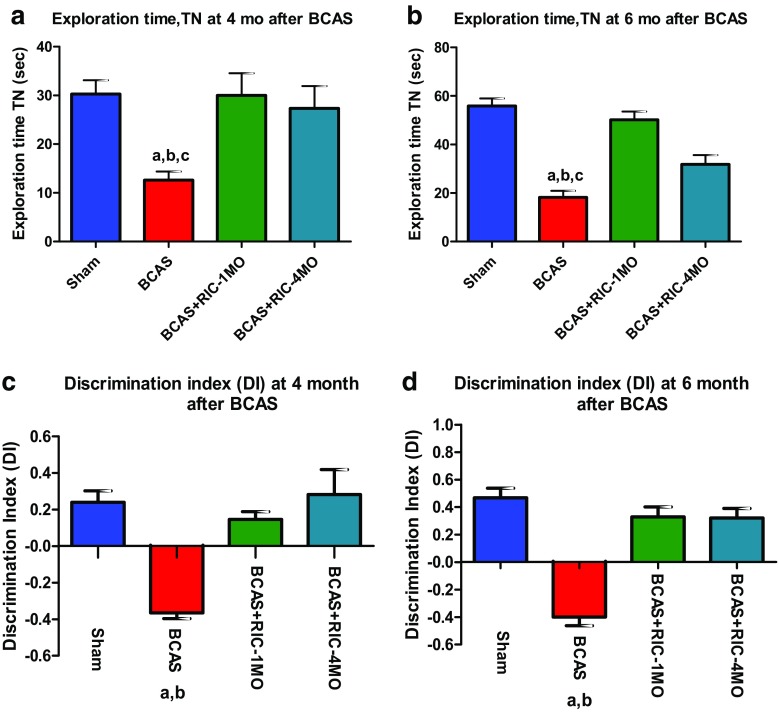
Using a repeated measures mixed model for time of exploration across (**a**) and (**b**),(**a**) time of exploration (TN) spent with the novel object at 4 months (4MO) after sham operation, BCAS, and BCAS+RIC with 1MO and 4MO. The RIC for 1MO and 4MO groups shows no significant difference but is significantly different than BCAS (sham RIC). Values are indicated as mean ± SE. ^a^ *p* < 0.001 vs sham; ^b^ *p* < 0.001 vs BCAS+RIC-1MO; ^*c*^ *p* < 0.01 vs BCAS+RIC-4MO. (b) TN spent with the novel object at 6 months (6MO) after sham operation, BCAS, and BCAS+RIC with 1MO and 4MO. Values are indicated as mean ± SE. ^a^ *p* < 0.001 vs sham; ^b^ *p* < 0.001 vs BCAS+RIC-1MO; ^c^ *p* < 0.01 vs BCAS+RIC-4MO. Using a repeated measures mixed model for the discrimination index across (**c**) and (**d**), (**c**) the discrimination index (DI) at 4 months (4MO) after sham operation, BCAS, and BCAS+RIC with 1MO and 4MO.The BCAS+RIC with 1MO and 4MO groups show no significant difference from one another but both are significantly different from the BCAS sham RIC. Values are indicated as mean ± SE. ^a^ *p* < 0.0001 vs sham; ^b^ *p* < 0.0001 vs BCAS+RIC-1MO and BCAS+RIC-4MO. (**d**) The discrimination index (DI) at 6 months (6MO) after sham operation, BCAS, and BCAS+RIC with 1MO and 4MO. There is no difference between 1MO and 4MO RIC groups, but both are significantly different than BCAS (sham RIC). Values are indicated as mean ± SE. ^a^ *p* < 0.0001 vs sham; ^b^ *p* < 0.0001 vs BCAS+RIC-1MO and BCAS+RIC-4MO

### RIC Improved Cognition Through Enhanced Spatial and Working Memory

Both 1MO and 4MO RIC improved cognition. Results from NOR test (for spatial memory) (Fig. 2a–d) and Y-maze test for working memory (Supplemental Fig. 3A–D) showed that the sham group is more attracted toward novel objects compared to BCAS. The BCAS group had significantly shorter exploration time for novel object (TN) and less discrimination index (DI). This indicates that BCAS causes impairment of discriminative ability in mice. RIC therapy for either 1-MO or 4-MO significantly restored the TN and DI scores. Moreover, RIC therapy for either 1MO or 4-MO significantly increased the entries’ alternations in the arms of Y-maze compared to the sham group tested at 4 and 6 months after BCAS.

**Fig. 3:**
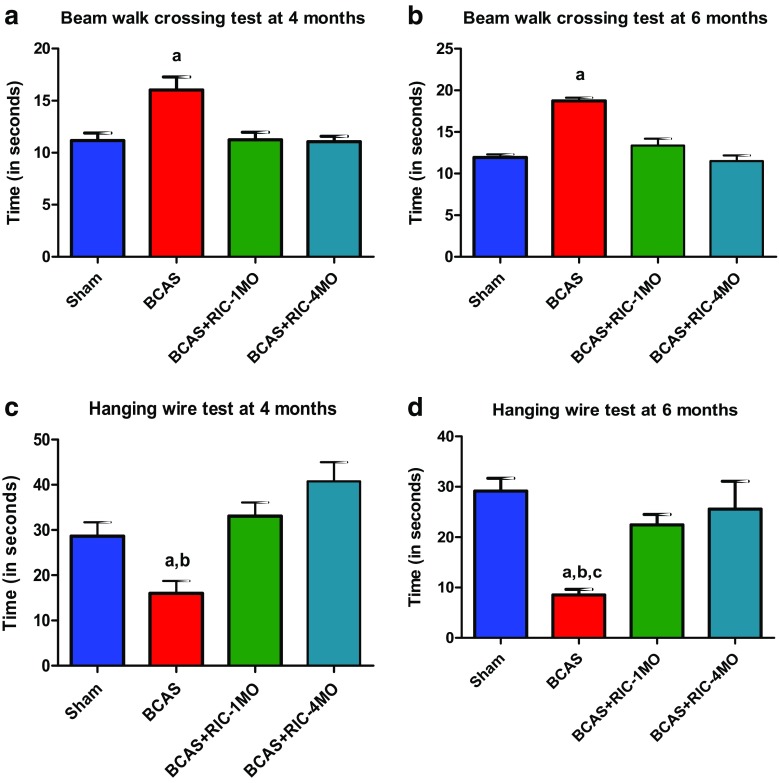
RIC prevents motor/muscular impairment after BCAS. Using a repeated measures mixed model for beam walk across (**a**) and (**b**), (**a**) time of crossing on a beam at 4 months (4MO) after sham operation, BCAS, and BCAS+RIC with 1MO and 4MO. Values are indicated as mean ± SE. ^a^ *p* < 0.0001 vs sham, BCAS+RIC-1MO, and BCAS+RIC-4MO. (**b**) Time of crossing on a beam at 6 months (6MO) after sham operation, BCAS, and BCAS+RIC with 1MO and 4MO. Values are indicated as mean ± SE.^a^ *p* < 0.0001 vs sham, BCAS+RIC-1MO, and BCAS+RIC-4MO. Using a repeated measures mixed model for the hanging wire test across (**c**) and (**d**), (**c**) cord-wire hanging test for muscular impairment at 4 months (4MO) after sham operation, BCAS, and BCAS+RIC with 1MO and 4MO. Values are indicated as mean ± SE. ^a^ *p* = 0.0008 vs sham; ^b^ *p* < 0.0001 vs BCAS+RIC-1MO and BCAS+RIC-4MO. (**d**) Cord-wire hanging test for muscular impairment at 6 months (4MO) after sham operation, BCAS, and BCAS+RIC with 1MO and 4MO. There is no difference between 1MO and 4MO RIC groups, but both significantly are different than BCAS (sham RIC). Values are indicated as mean ± SE. ^a^ *p* < 0.0001 vs sham; ^b^ *p* = 0.0008 vs BCAS+RIC-1MO; ^c^ *p* = 0.0001 BCAS+RIC-4MO

### RIC Improved Muscular/Motor Function

Animals subjected to BCAS spent more time to cross a beam in comparison to the sham control animals (Fig. 3a, b). RIC therapy for either 1MO or 4MO significantly reduced the time spent by the animals to cross the beam as compared to BCAS groups. Moreover, muscular impairment or motor impairment was assayed by wire hanging test (Fig. 3c, d). BCAS groups spent significantly less time suspending their body to the hanging wire compared to sham. RIC therapy for 1MO or 4MO significantly increased the hanging wire time compared to the BCAS group.

### RIC Enhanced Angiogenesis and Arteriogenesis

**Fig. 4:**
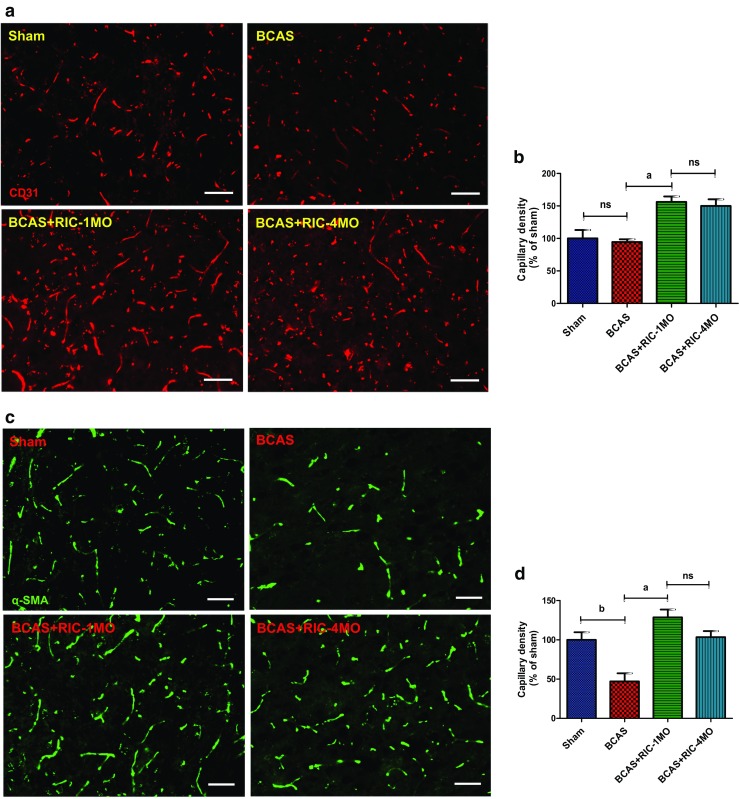
RIC promotes angiogenesis and arteriogenesis. **a**, **c** Representative photomicrographs of single immunofluorescence for CD31 (*red*, (**a**)) and α-SMA (*green*, (**c**)) or double immunofluorescence (Supplemental Figs. 4 and 5 for CD31 and α-SMA with DAPI) for vessels in the striatum (caudoputamen) of each indicated group at 6 months with or without RIC therapy (*scale bar* for CD31 and α-SMA = 20 μm/20×). **b**, **d** The quantitative analysis shows of capillary density at 6 months in each indicated group (*N* = 4 to 6/group; ^b^ *p* < 0.01 vs sham; ^a^ *p* < 0.001 vs BCAS+RIC-1M/4MO)

RIC therapy significantly increased capillary density, angiogenesis, and arteriogenesis as indicated by increased expression of CD31 and α-SMA, compared to the BCAS group (Fig. 4a–d; Supplemental Figs. 4 and 5). However, there was no significant difference between RIC-1MO and RIC-4MO therapy groups. Moreover, RIC therapy increased the expression and colocalization of pericytes with cerebral blood vessels as indicated by increased expression of PDGFR-B and IB4, respectively (Supplemental Fig. 6A). Chronic treatment also showed to increase the capillary diameter (Supplemental Fig. 6B).

**Fig. 5:**
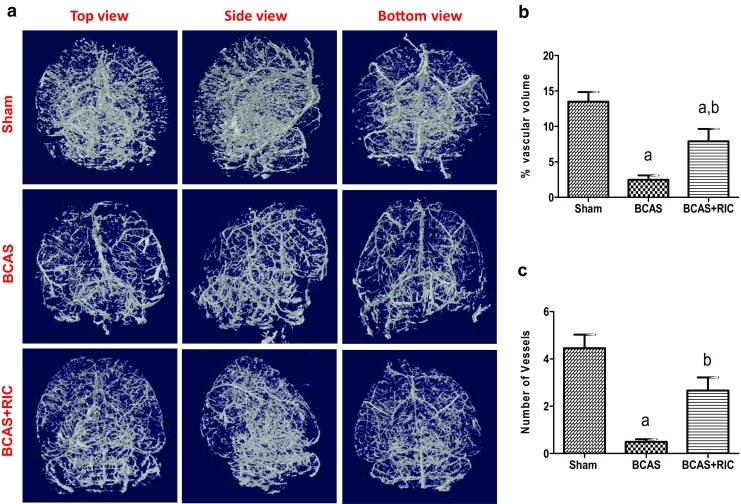
RIC facilitates cerebrovascular angioarchitecture with 3 weeks treatment after BCAS. (**a**) Representative 3D images showing the whole cerebrovascular angioarchitecture from a top, side, and bottom view of sham, BCAS, and BCSA+RIC groups of mice brain. Histogram showing vascular volume percentage (**b**) and number of vessels (**c**) for linear space between vessels, density, and lumen thickness (Supplemental Fig. 9A–C). *N* = 8/group, ^a^ *p* < 0.01 vs sham; ^b^ *p* < 0.05 vs BCAS

**Fig. 6:**
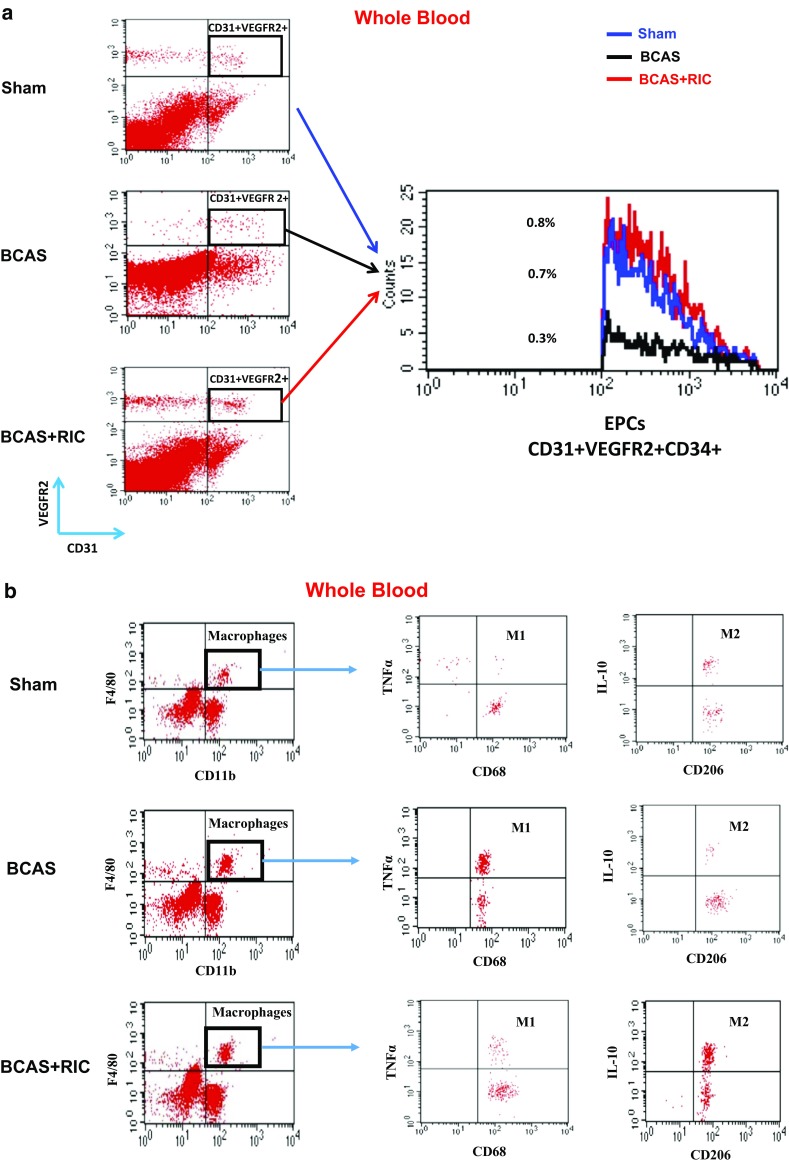

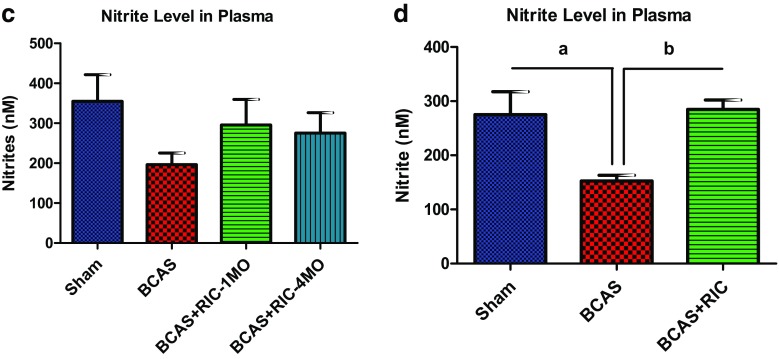
RIC therapy activates endothelial progenitor cells (EPCs) and increases M2/M1 macrophages in the blood 3 weeks treatment. (**a**) Flow cytometry graphs showing a significant increase the EPCs count, as indicated by increased expression of CD31, VEGFR2, and CD34. (**b**) Dropped CBF in BCAS group results in recruitment of macrophages in response to chronic ischemia. RIC therapy decreased the level of inflammatory M1 macrophages while it enhanced the level of anti-inflammatory M2 macrophages in the blood (as indicated by the expression of CD11b, F4/80; CD68, TNFα; and CD206, IL-10). However, a high level of circulating M1 macrophage was counted in BCAS groups, indicating high inflammatory burden. RIC therapy activated circulating EPCs, thus reduced the vascular injury and protects ischemic brain. (**c**) RIC therapy with 1-MO or 4-MO showed a trend but insignificant increase in plasma nitrite levels at 6 months compared to BCAS group. However, RIC therapy for 3 weeks post BCAS significantly increased the plasma nitrite levels compared to BCAS sham RIC (**d**). Values are indicated as mean ± SD. ^a^ *p* = 0.0092 vs sham; ^b^ *p* = 0.0044 vs BCAS+RIC

### RIC Reduced White Matter Damage and Myelin Basic Protein (MBP)

The white matter degeneration in the corpus callosum was tested by Klüver–Barrera staining. The intensity in Klüver–Barrera staining was significantly reduced after BCAS compared to sham animals. RIC therapy for 1 or 4 months significantly reversed the BCAS-induced white matter damage (Supplemental Fig. 7A–B). MBP staining significantly decreased in cortical and hippocampal CA1 field region (Supplemental Fig. 8A–B), and this was reversed with RIC therapy in both BCAS+RIC1-MO and BCAS+RIC4-MO groups.

### RIC Improves the Cerebrovascular Angioarchitecture and Increases NO Production

The use of BriteVu staining enabled the 3D visualization of the complete tree of the cerebrovasculature. BCAS caused a significant decline in number and volume of cerebral vessels. RIC significantly induced angiogenesis and collaterals formation as indicated by the increase in the vessels number and volume (Fig. 5a–c; Supplemental Fig. 9A–C). In support with this, results from the flow cytometry studies showed a corresponding increase in the EPC count (as indicated by increased expression of CD31 and VEGF-R2) and in macrophage expression and polarization (as indicated by increased expression of CD11b, F4/80, and CD206) with RIC therapy (Fig. 6a, b).

RIC therapy for 3 weeks post BCAS significantly increased the plasma nitrite levels compared to BCAS without RIC. However, RIC therapy for 1-MO and 4-MO showed a trend but insignificant increase in plasma nitrite levels at 6 months compared to the BCAS group (Fig. 6c, d). However, plasma nitrite level in the BCAS group was significantly decreased as compared to the sham group. Probably the short half-life of NO and long delay after RIC was finished caused NO levels to decrease.

## Discussion

RIC can be performed before acute cerebral ischemia (preconditioning), during acute ischemia (per-conditioning), or after reperfusion (post conditioning). There is a large body of evidence that acute remote ischemic conditioning is a powerful cerebroprotectant in acute focal cerebral ischemia models [27, 28]. The current study proposes C-RIC as a therapeutic paradigm in chronic mild ischemia. The repetitive use of RIC for weeks or months can be an analogue to long-term daily exercise. There are fewer published studies and less data on C-RIC in chronic cerebral ischemia. C-RIC administered for 6 months was effective in reducing recurrent stroke and TIA in two small randomized clinical trials in patients with intracranial atherosclerosis (ICAS) [29, 30]. There was increased CBF by SPECT in one trial suggesting that C-RIC increased CBF in human patients. There is also a small clinical trial suggesting that C-RIC may reduce progression of white matter disease in patients with VCID. However, we have little preclinical data to support its use clinically, or to better understand its mechanisms. The BCAS mouse model has been offered as a model to test interventions in cerebral small vessel disease and VCID, and we now describe findings that support translation to the bedside.

First, our results show that both 1 month C-RIC (1-MO) and 4 months of C-RIC (4-MO) are equally effective in improving long-term (6 months) CBF, working and spatial memory, and in improving balance and motor skills compared with sham animals. This is a crucial piece of data suggesting that shorter term use of C-RIC (1 month) in patients with chronic ischemia may be as effective as longer term treatment (4 months). However, we exercise caution in extrapolating these findings to human VCID/small vessel disease, as the BCAS model has a known start and onset time. In VCID patients, the course of disease is insidious and there is no precise start time. Nevertheless, in our preclinical model, C-RIC appears to be efficient in improving the long-term CBF and long-term cognitive and motor function, likely by inducing angiogenesis and collateral remodeling. Our findings may have some implications to use C-RIC in ICAS where there is hypoperfusion, albeit from intracranial rather than extracranial stenosis. While the clinical trials in ICAS have used 6 months or 300 days of C-RIC, our results show that shorter durations may be effective.

Second, C-RIC for 3 weeks induces cerebral vascular remodeling, angiogenesis, arteriogenesis, and new collateral formation. These structural changes in the angioarchitecture may underlie the improvement in CBF. Beyond the functions of supplying oxygen and nutrients, endothelial cells have trophic functions for neurons and oligodendrocytes. This angiogenesis and improved CBF are likely protective to oligodendrocytes and neurons. This increase in cerebral angiogenesis is similar to what is seen in the rodents’ models of daily physical exercise for 3–4 weeks [31–34]. There are strong parallels between RIC and physical exercise. Both involve shear stress to the vasculature and upregulation of endothelial NOS (eNOS) and increased plasma nitrite [35, 36].

Third, similar to exercise, C-RIC increases circulating EPCs, which may be playing a role in angiogenesis. While there is controversy over whether EPCs incorporate directly into growing vessels or provide trophic effect to vessels, they are associated with angiogenesis and vascular health. Further studies are needed to define their participation in angiogenesis in our model.

Fourth, C-RIC is associated with increases in plasma nitrite, which suggests a role for increased NO bioavailability in mediating vascular remodeling. While once regarded as an inert molecule and oxidative end product of NO metabolism, research over the last decade has shown that *nitrite* serves a “storage” pool of NO derived from endogenous eNOS [37–39]. Nitrite circulates in the blood associated with RBC/hemoglobin and is reduced to NO in areas of hypoxemia, mediating hypoxic vasodilatation [38, 40]. While NO is normally limited to a paracrine effect due to its very short half-life, the eNOS/nitrite/NO system provides a distant “endocrine” effect of NO. We showed in this study that increased NO bioavailability is associated with angiogenesis and collateral remodeling. Moreover, NO is associated with mobilization of endothelial progenitor cells.

We have demonstrated that C-RIC is effective in promoting angiogenesis and improving long-term functional outcome. The major risk factor for dementia and VCID is age. It is paramount to demonstrate these findings in aged mice of both sexes, and we are conducting series of these studies in our group. C-RIC is a safe and well-tolerated intervention that may be useful in patients with VCID. Further studies are needed to determine the dosing and to develop biomarkers. Both EPCs and plasma nitrite may serve as useful biomarkers.

## Electronic Supplementary Material

ESM 1 (PDF 5671 kb)

## Abbreviations

1MO: One month
4MO: Four months
6MO: Six months
BCAS: Bilateral carotid artery stenosis
CCAs: Common carotid arteries
CBF: Cerebral blood flow
RIC: Remote ischemic conditioning
C-RIC: Chronic remote ischemic conditioning
GFAP: Glial fibrillary acidic protein
IF: Immunofluorescence
IHC: Immunohistochemistry
LSCI: Laser Speckle Contrast Imager
MBP: Myelin basic protein
NO: Nitric oxide
VCID: Vascular contributions to cognitive impairment and dementia
WM: White matter
