# Supplementary material for: Chronic Remote Ischemic Conditioning Is Cerebroprotective and Induces Vascular Remodeling in a VCID Model
Source: Transl Stroke Res. 2017 Jul 28;9(1):51–63. doi: 10.1007/s12975-017-0555-1 (PMC5750336; doi:10.1007/s12975-017-0555-1)
Supplement: Supplementary file 1 — (PDF 5671 kb) [file 12975_2017_555_MOESM1_ESM.pdf]

ONLINE SUPPLEMENT

Chronic Remote Ischemic Conditioning is Cerebroprotective and Induces Vascular Remodeling in a VCID Model

Mohammad Badruzzaman Khan,* PhD, Sherif Hafez PhD, Md. Nasrul Hoda PhD, Babak Baban PhD, Jesse Wagner BS, Mohamed E Awad MD, Hasith Sangabathula BS, Stephen Haigh BS, Mohammed Elsalanty PhD, Jennifer L. Waller[5] PhD and David C Hess* MD

Contents:

1. Supplemental Methods
2. Supplemental Figure with legends

*Address for Co-Correspondence:

Mohammad Badruzzaman Khan, PhD

David C. Hess, MD

1120 15th Street, CA 1053

Department of Neurology, Medical College of Georgia, Augusta University, Augusta, Georgia 30912, USA

Email: mkhan@augusta.edu

Email: dhess@augusta.edu

Telephone: +1-706-721-1698/1691

Fax: +1-706-721-7619

Supplemental Methods

Bilateral carotid artery stenosis (BCAS) Surgery

Bilateral Common Carotid Artery Stenosis (BCAS) procedure in the mouse was performed as described elsewhere [1, 2]. Briefly, mice were subjected to BCAS and subsequent cerebral chronic hypoperfusion using microcoils specially designed for the mouse (microcoil specifications: piano wire diameter 0.08mm, internal diameter 0.18mm, coiling pitch 0.5mm, and total length 2.5mm; Sawane Spring Co Ltd, Japan). To induce VCID in the Buprenex sedated mice under isofluorane placed on a thermoregulated bed to maintain body temperature at 37 ºC during surgery; a midline cervical skin incision was made ventrally. The two common carotid arteries (CCA) were exposed one-by-one, freed from their sheaths, and a microcoil was twined by rotating it around the each CCA, as published elsewhere. The site of surgery was closed, and mice were observed and taken care of post-surgery until conscious and recovered to freely access food and water ad libitum. In Sham group, the skin was incised and the two common carotid arteries were exposed one by one.

Non-Invasive RIC Therapeutic methods

Non-invasive RIC therapy using a BP-cuff was performed as reported earlier [3, 4] with certain modifications by us [2]. An automated blood pressure instrument (SC1000 rodent BP instrument remodeled as a RIC-BP dual mode instrument with software and computer) was modified with a multi-channel RIC device for multiple rodents (RC2000, V1.14; a customized product developed by Hatteras Instrument; Cary NC, for our laboratory) to produce cuff-based, non-invasive, bilateral RIC in the mouse hind limbs. After 1 week post-BCAS surgery, RIC was started daily. RIC was performed in isoflurane anesthetized mice with the modified instrument.. Briefly, mice were placed on a thermoregulated bed to maintain body temperature at 37 ºC and slightly stretched limbs were secured using paper tape. Customized mouse limb cuffs were wrapped on each hind limb and RIC was performed as 4 cycles x 5 minutes/cycle at 200 mmHg using a 5-min inflate and deflate in each cycle. Sham RIC mice were secured in the same fashion, but the BP cuff was not inflated, controlling for any effects of stress or handling. Following the RIC therapy, mice were placed into a clean home cage and monitored until conscious.

Estimation of nitrite in plasma

Plasma NOx ($NO2^-$ + $NO3^-$) levels were measured by NO-specific chemiluminescence, as described previously [5] with slight modification. Blood were collected from each groups of mice in isoflurane anesthetized condition with cardiac puncture. The plasma sample were prepared by centrifuging the blood sample for 20 min at 2000 rpm / 4 ºC. Plasma (100 ul) was mixed with twice volumes of ethanol (100%) and kept at -20 C for 40-60 min followed by

centrifuged at maximum rpm (13000 rpm) for 10 min to remove protein as pellet. The supernatant (100 ul) was taken and injected to measure nitrite. The nitrite levels were measured by NO Analyzer 280i (GE Analytical instruments, Colorado, USA). The level of nitrite was expressed in nM.

Behavioral test: Functional outcomes and cognitive test

Novel object test

Behavioral assessment by novel object recognition (NOR) test was performed as reported earlier by us [2, 6]. The 2-trial novel object recognition task was also performed in which a mouse was placed in an enclosed box (40 inch x 40 inch) with 2 identical objects situated within a 4-inch diameter circle and located a set distance apart. The mouse was then removed from the environment for a set amount of time and 1 of the 2 previously used (familiar) objects was replaced with a novel object that was different from the familiar object in texture and appearance. The mouse's behavior on exposure to the novel object was then recorded. This test is based on the natural tendency of mice to investigate a novel object rather than a familiar one, which reflects the use of learning and recognition memory processes. The capability of the mouse to discriminate between a familiar versus novel object was determined as the discrimination index, DI= (Tn-Tf)/ (Tn+Tf), where Tn is the time spent by the mouse with the novel object and Tf indicates the time spent with the familiar object.

Wire hanging test

This test was used as a measure of grasping ability, forelimb strength and coordination movements described earlier [7]. Mice used their forelimbs to suspend their body on a single 1 mm diameter cord stretched mid-way between two metal stands/post (55 cm long suspended horizontally, 50 cm above a standard mouse cage was filled with soft cloths and under the cord in between the stands).

The latency to fall was measured five times (in seconds) for each mouse. The first two trials served to familiarize the mouse with the testing conditions. The subsequently three trials were taken at five minute intervals to allow a recovery period. The latency time measurements began from the point when the mouse was hanging free on the wire and ended with the animal falling to the cage underneath the cord. No attempt was made to force compliance during any of the various trials. If an animal adopted a strategy that permitted an extended hanging time, this was allowed and the true latency to fall time was recorded (in sec). In cases where an animal was unable to hang from the wire for at least one second, a fall time of zero was noted for that trial.

Beam Walk test

Motor balance and coordination test was performed by using the balance beam as reported earlier [8] with a little modification. The beam apparatus consists of 1.25 meter beams (scale marked) with a flat surface of 6 mm width) resting 20 cm above the table top on two poles. A

black box is placed at the end of the beam as the finish point. (Plywood and other types of wood for the beams, poles, and box can be used). Nesting material from home cages is placed in the black box to attract the mouse to the finish point. A lamp (with 60 watt light bulb) is used to shine light above the start point and serves as an aversive stimulus. Approximately 10-15 min prior to training/testing, the mice can be transported to the room to acclimatization where the beam apparatus are located. Mice were trained before the real test for three times. During training, mice were encouraged to keep moving across the beam by prodding, poking, or pushing it from behind with gloved fingers. Training trials were repeated until each animal crossed the beam three times without stopping or turning around. On a final testing, three trials were performed per mouse to cross a beam. The time (in seconds) to cross the beam (100 cm) is counted by timer (one at 25 cm that starts a timer and one at 100 cm that stops the timer). A soft bed /cloths is stretched below the beam above the table top, to cushion any falls. Trials in which the animal stopped or turned around were repeated. The average of the trials was calculated in seconds.

Y-maze test

A continuous spontaneous alternation test was performed at 4 and 6 months in all groups after the sham surgery and BCAS surgery using a Y-maze (San Diego Instruments, San Diego, CA) as described previously [9]. The Y-maze consisted of three arms at 120∘ and was made of beige plastic. Each arm was 7.5 cm wide and 38 cm long, and its three sides (except for the side adjoining the other arms) were surrounded by 12.5-cm high walls. The floor of the Y-maze was covered with a sawdust bedding material. Between each trial, the sawdust was mixed and redispersed to remove or randomize odor trails. Distal visual cues were placed around the Y-maze. A mouse was placed in one arm and allowed to explore for 7 min freely. Mouse behavior was monitored, recorded, and analyzed by a webcam (C920, Logitech, Newark, CA) and the Any-Maze software (Stoelting, Wood Dale, IL). A mouse was considered to have entered an arm if the whole body (except for the tail) entered the arm and to have exited if the whole body (except for the tail) exited the arm. If an animal consecutively entered three different arms, it was counted as an alternating triad. Because the maximum number of triads is the total number of arm entries minus 2, the score of alternation was calculated as "the number of alternating triads/ (the total number of arm entries minus 2)."

Histological and Immunohistochemical Assessment

For histological and immunohistochemical staining, a standard paraffin block and frozen block were used to get the coronal sections of thickness 6 um and 10 um respectively. Each slide was mounted with 4 sections. Histochemical-staining for Fiber density of Luxol-Fast-blue (LFB, a demyelination marker)-neutral red staining was performed to detect the severity of WM lesion [2, 10, 11] with slightly modification. Briefly, de-waxed rehydrated sections were immersed in the LFB solution (Solvent Blue 38, Sigma) at 60 °C overnight. Excess stain was removed by 95% ethanol treatment followed by washing with deionized water. Grey and white matter differentiation was initiated with the treatment of 0.05% aqueous lithium carbonate (Sigma) for 30 second followed by 70% ethanol until the nuclei are decolorized. Sections were immersed in neutral red solution (Sigma) for 30 min and washed in deionized water. They were dehydrated in ethanol gradient (70 – 100%), and finally cleared in xylene and mounted with

cytoseal. For immunostaining, mice brain sections were deparaffinized and pretreated with citrate buffer (pH 6.0) for antigen retrieval at 120 C followed by cooling (30 min at room temperature) and subsequently washed with PBS twice. The sections were incubated with anti-MBP (C-16 clone, SC-13914, Santa Cruz, CA, USA; 1:100 dilutions) overnight at 4 C, followed by incubation with appropriate biotinylated secondary antibody (Vector Laboratories, Burlingame, CA; 1:200 dilution) for 1 hour. To visualize the immunoreactivity, sections were finally incubated with an avidin–biotin peroxidase complex solution (Vectastain ABC kit, Vector Laboratories, diluted 1:100) for 30 min, followed by diaminobenzidine (DAB) as the substrate (Vector Laboratories). After staining, the sections were counterstained with Harris hematoxylin (cat. #HHS35-1L; Sigma) for few seconds. The sections were then dehydrated rapidly through ethanol and xylene, and mounted with VectaMount medium (Vector). Images were captured with Zeiss microscope integrated with Axio Vision software.

For immunofluorescence staining, frozen sections of mouse brain were air dried for 20 min prior to acetone fixation at -20 C for 5 min and washed immediately twice in PBS with 0.05% Tween-20. The sections were then blocked with 5% goat serum in PBS with 0.1% Tween-20 for 1 hr at room temperature. The section were first labeled with rat anti-platelet endothelial cell adhesion molecule [PECAME-1(CD31), BD # 550274, USA,1:200 dilution], mouse monoclonal anti-α-Smooth muscle actin [α-SMA, SC-53142, Santa Cruz, CA, USA; 1:50 dilution] and anti-platelet- derived growth factor [PDGFR-β (958): SC-432, Santa Cruz, CA USA; 1:200 dilution] overnight at 4 C. After washing, the sections were labeled with Alexa Fluor 594-conjugated anti-rat IgG for CD31 (1:500), Alexa Fluor 488-conjugated anti-mouse IgG for α-SMA (1:500), Alexa Fluor 594-conjugated anti-rabbit IgG for PDGFR-β (1:500), for 1 h at room temperature and Isolectin B4 conjugates (Invitrogen, molecular probe Life Technology , IB4 # 121411) for 3 h at room temperature. These secondary antibodies were purchased from Molecular Probes (Eugene, OR, USA). The sections were then mounted with DAPI fluoromount-G® (SouthernBiotech) and fluoromount solution (Sigma). Finally, the slides were analyzed by an Axio Imager M1 microscope (Zeiss, Thornwood, NY, USA). The localization of CD31 and PDGFR-B was shown by the red fluorescence of Alexa fluor 594 and α-SMA was shown by the green fluorescence of Alexa fluor 488. The capillary number/density was quantified by the number of CD31 and α-SMA positive cells (cells/mm2) and were examined by Image J software (NIH, USA) in striatum (caudoputamen) from the 5 images or slices of 5 fields at similar coronal section of each animal (n=4-5). The percentage or the cell numbers were corrected by the analyses size and normalized to those found in the sham groups. The severity of white matter (WM) lesion was graded into four levels on the basis of Klüver-Brrera (Luxol Fast Blue) staining by an investigator blind to the experimental condition as no stain (0), low stain (1), moderate stain (2) and high stain (3).

Supplementary References:

1. Shibata M, Ohtani R, Ihara M, Tomimoto H. White matter lesions and glial activation in a novel mouse model of chronic cerebral hypoperfusion. Stroke. 2004;35:2598-2603

2. Khan MB, Hoda MN, Vaibhav K, Giri S, Wang P, Waller JL et al. Remote Ischemic Postconditioning: Harnessing Endogenous Protection in a Murine Model of Vascular Cognitive Impairment. Transl Stroke Res. 2015; 6(1):69-77. doi:10.1007/s12975-014-0374-6.

3. Røpcke DM, Hjortdal VE, Toft GE, Jensen MO, Kristensen SD. Remote ischemic preconditioning reduces thrombus formation in the rat. J Thromb Haemost. 2012; 10(11):2405-6. doi: 10.1111/j.1538-7836.2012.04914.x.

4. Yuan HJ, Zhu XH, Luo Q, Wu YN, Kang Y, Jiao JJ, et al. Noninvasive delayed limb ischemic preconditioning in rats increases antioxidant activities in cerebral tissue during severe ischemia reperfusion injury. J Surg Res. 2012; 174:176–83.

5. Fulton D, Harris MB, Kemp BE, Venema RC, Marrero MB, Stepp DW. Insulin resistance does not diminish eNOS expression, phosphorylation, or binding to HSP-90. Am J Physiol Heart Circ Physiol. 2004;287(6):H2384-93. doi:10.1152/ajpheart.00280.2004.

6. Wakade C, Sukumari-Ramesh S, Laird MD, Dhandapani KM, Vender JR. Delayed reduction in hippocampal postsynaptic density protein-95 expression temporally correlates with cognitive dysfunction following controlled cortical impact in mice. Journal of neurosurgery. 2010; 113:1195-1201

7. Klein SM, Vykoukal J, Lechler P, Zeitler K, Gehmert S, Schreml S et al. Noninvasive in vivo assessment of muscle impairment in the mdx mouse model--a comparison of two common wire hanging methods with two different results. J Neurosci Methods. 2012 Jan 30; 203(2):292-7.

8. Luong TN, Carlisle HJ, Southwell A, Patterson PH. Assessment of motor balance and coordination in mice using the balance beam. J Vis Exp. 2011 Mar 10 ;(49).

9. Li Y, Kim J. CB2 Cannabinoid Receptor Knockout in Mice Impairs Contextual Long-Term Memory and Enhances Spatial Working Memory. Neural Plast. 2016; 2016:9817089.

10. Shibata M, Ohtani R, Ihara M, Tomimoto H. White matter lesions and glial activation in a novel mouse model of chronic cerebral hypoperfusion. Stroke. 2004; 35:2598-2603.

11. Fujita Y, Ihara M, Ushiki T, Hirai H, Kizaka-Kondoh S, Hiraoka M, Ito H, Takahashi R. Early protective effect of bone marrow mononuclear cells against ischemic white matter damage through augmentation of cerebral blood flow. Stroke. 2010; 41(12):2938-43.

Supplemental Figure 1.

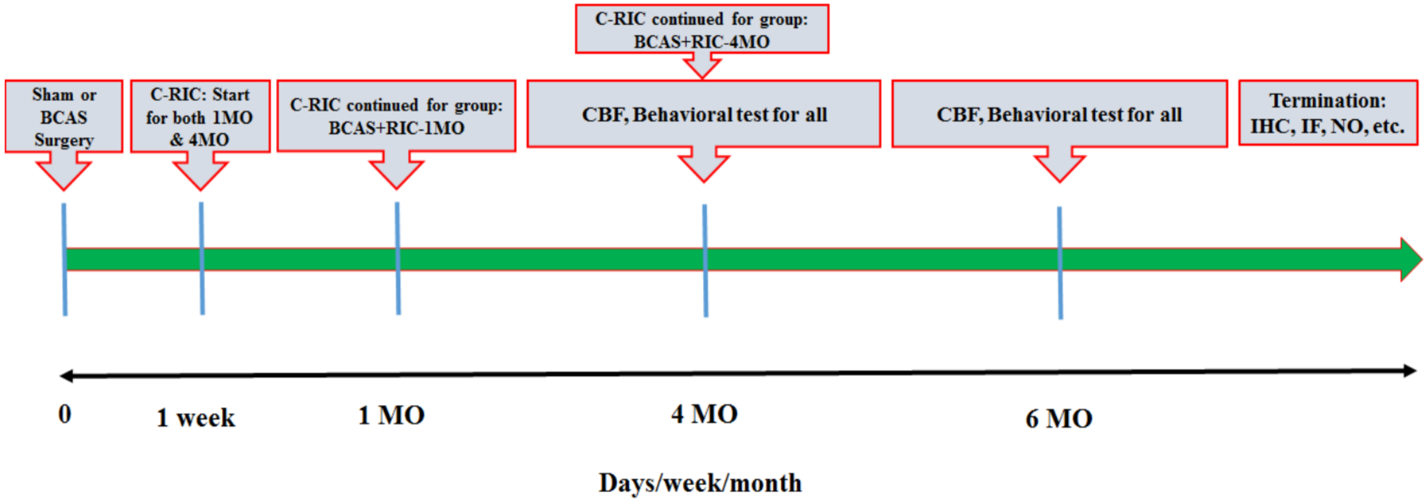

Supplemental Figure 1. Schematic representation in days/week/months of the experimental design. BCAS, Bilateral carotid artery stenosis; C-RIC, Chronic Remote Ischemic Conditioning; MO, month; CBF, cerebral blood flow; Day 0 indicates to the day of surgery (Sham or BCAS); IHC, immunohistochemical and histopathological studies; IF, immunofluorescence; NO, nitric oxide.

Supplemental Figure 2.

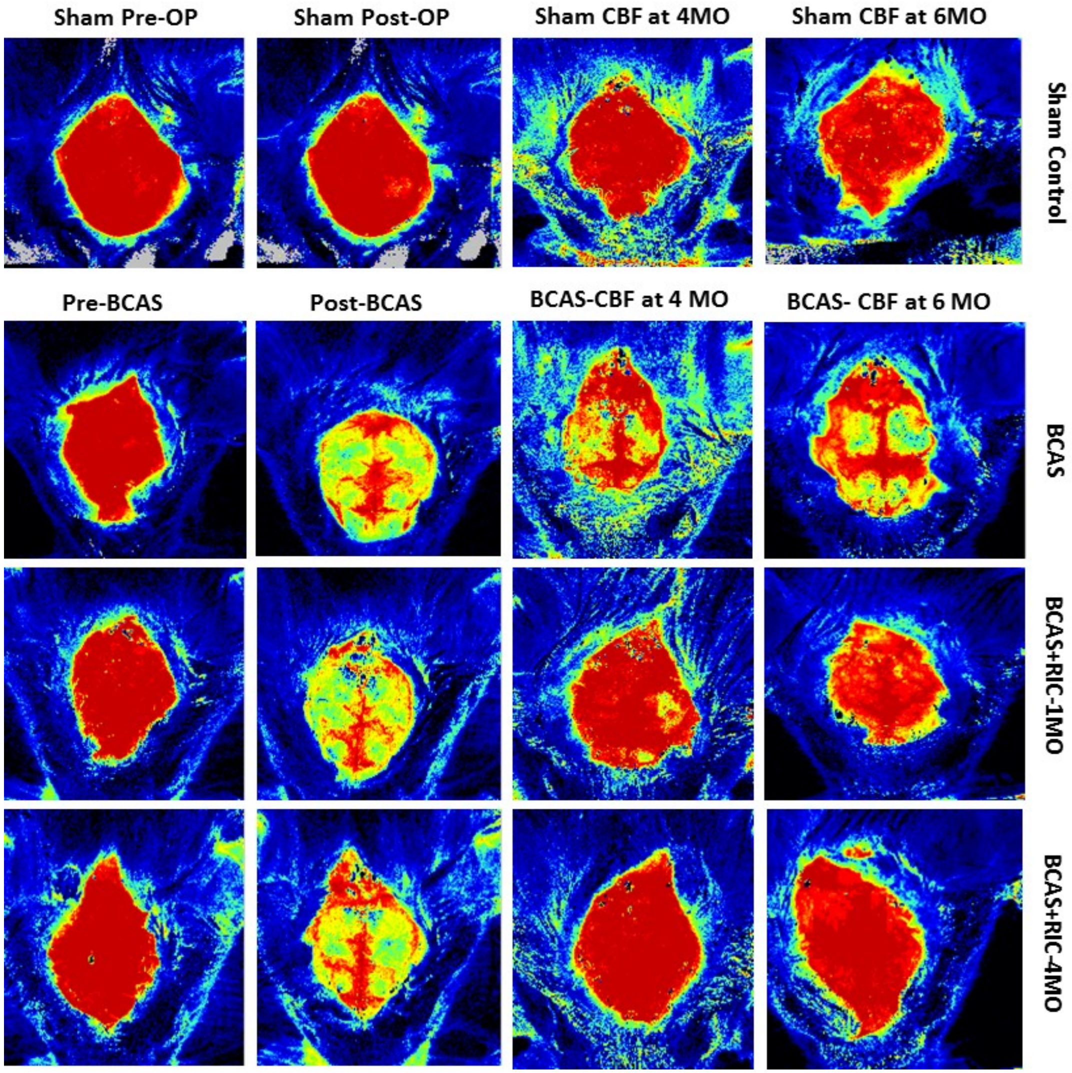

Supplemental Figure 2. Representative images showing cortical changes of CBF for Sham BCAS, BCAS (sham RIC), BCAS+RIC-1MO and BCAS+RIC-4MO at different time points including: Pre-, post-, at 4MO and 6MO after sham or BCAS surgery (see histogram Figure 1b-e for statistical analyses).

Supplemental Figure 3.

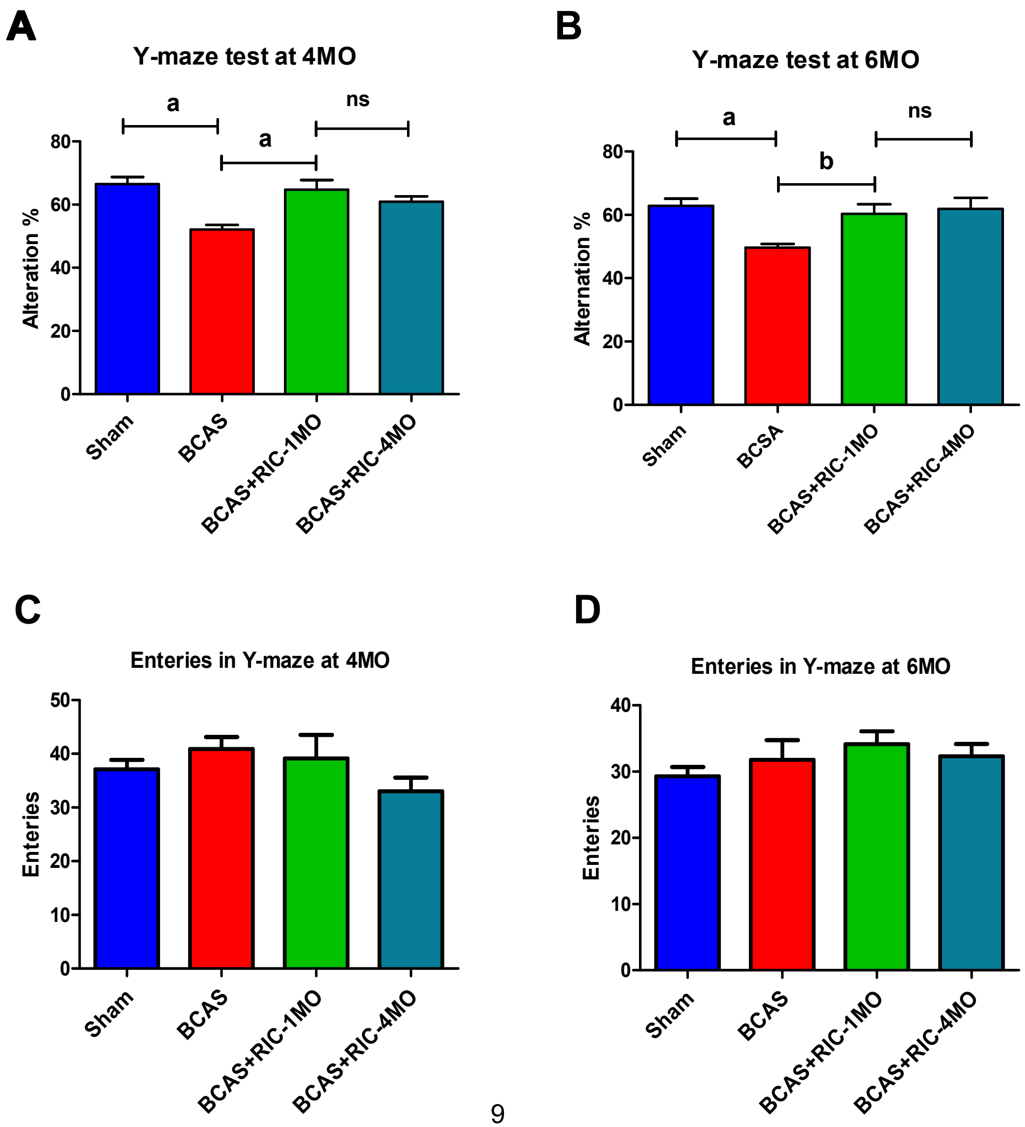

Supplemental Figure 3 (A-D). Representative images of Y-maze test. RIC prevents working memory deficits after BCAS. **A**, Histogram showing spontaneous alternation in the Y-maze test of groups Sham BCAS, BCAS + sham RIC, BCAS+RIC-1MO and BCAS+RIC-4MO at 4 month after sham or BCAS surgery (N= 7 to 10). Value are indicates as mean ±SE. $^{a}p<0.0001$ in Sham vs BCAS; $^{a}p<0.0001$ in BCAS vs BCAS+RIC-1MO and 4-MO and at 4 months. **B**, Histogram showing spontaneous alternation in the Y- maze test of groups Sham, BCAS, BCAS+RIC-1MO and BCAS+RIC-4MO at 6 month after sham or BCAS surgery (N= 7 to 10). Value are indicated as mean ±SE. $^{a}p<0.0001$ in Sham vs BCAS; $^{b}p=0.0007$ in BCAS vs BCAS+RIC-1MO and 4-MO and at 4 months. **C,** Histogram showing spontaneous activity in the Y maze test of groups Sham, BCAS, BCAS+RIC-1MO and p=0.0002 in BCAS+RIC-4MO at 4 month after sham or BCAS surgery (N=7 to 10). **D,** Histogram showing spontaneous activity in the Y maze test of groups Sham, BCAS, BCAS+RIC-1MO and BCAS+RIC-4MO at 6 month after sham or BCAS surgery (N= 7 to 10).

Supplemental Figure 4.

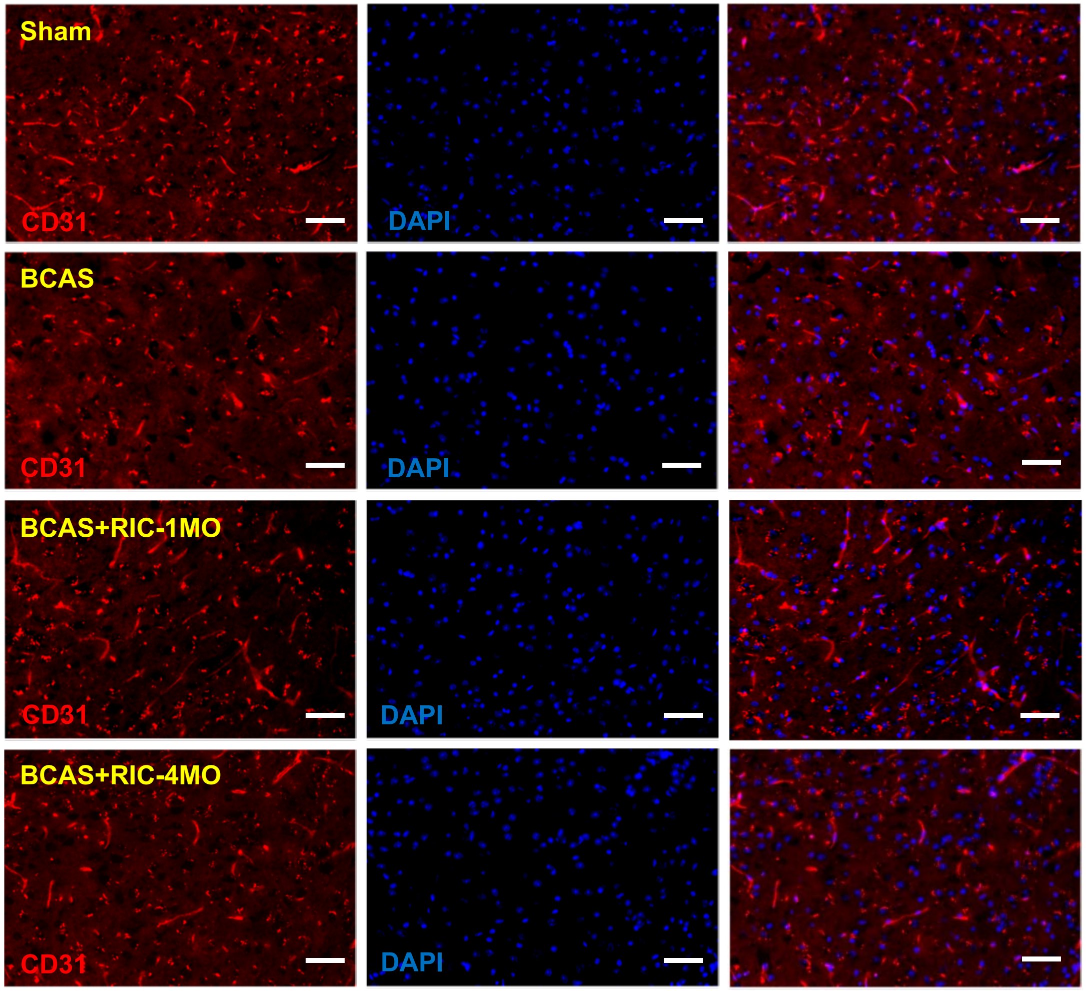

Supplemental Figure 4. Representative double immunofluorescence images of CD31 (red)/Dapi (blue) of the striatum (caudaputamen) region of all indicated groups showing after sham and BCAS surgery and increases their angiogenesis by RIC.

Supplemental Figure 5.

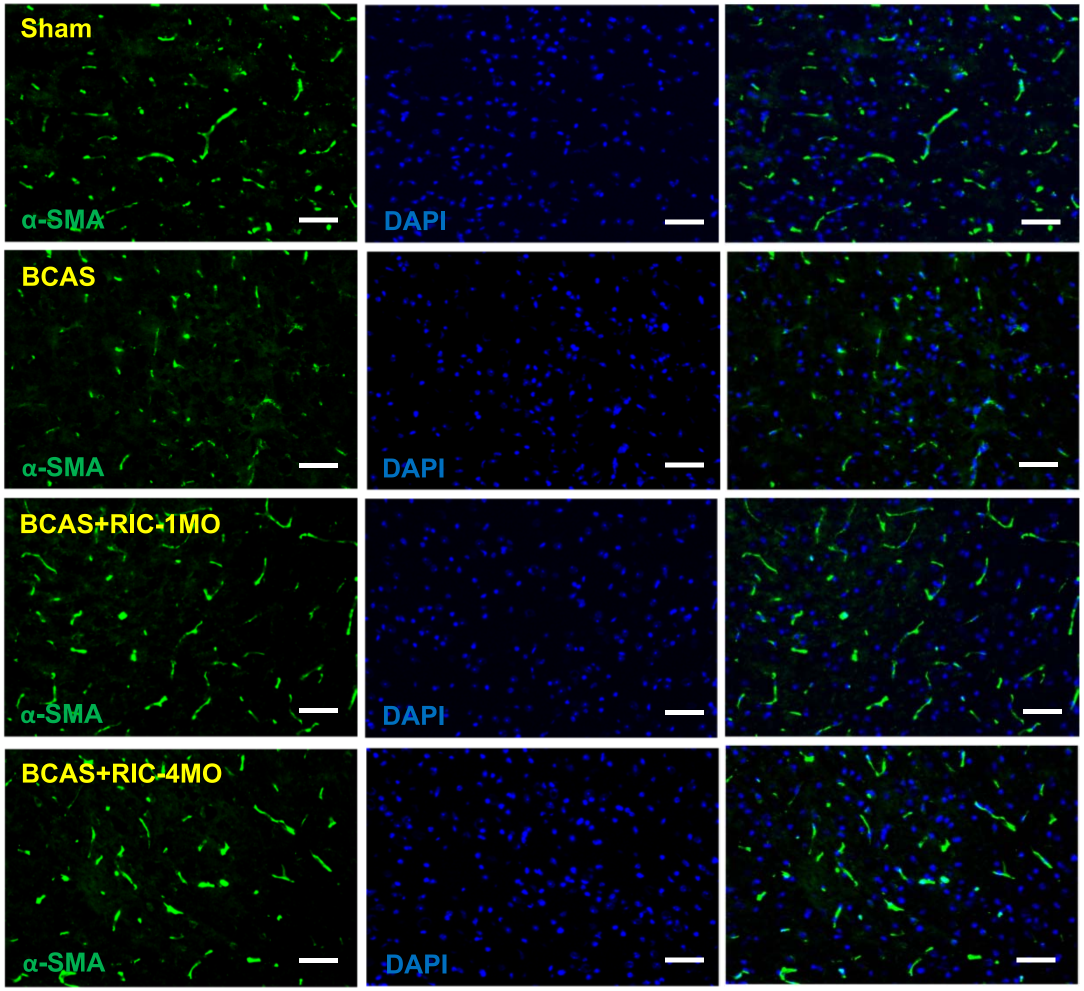

Supplemental Figure 5. Representative double immunofluorescence images of α-SMA (green)/Dapi (blue) of the striatum (caudaputamen) region of all indicated groups showing after sham and BCAS surgery and increases their collateral architecture or arteriogenesis by RIC.

Supplemental Figure 6.

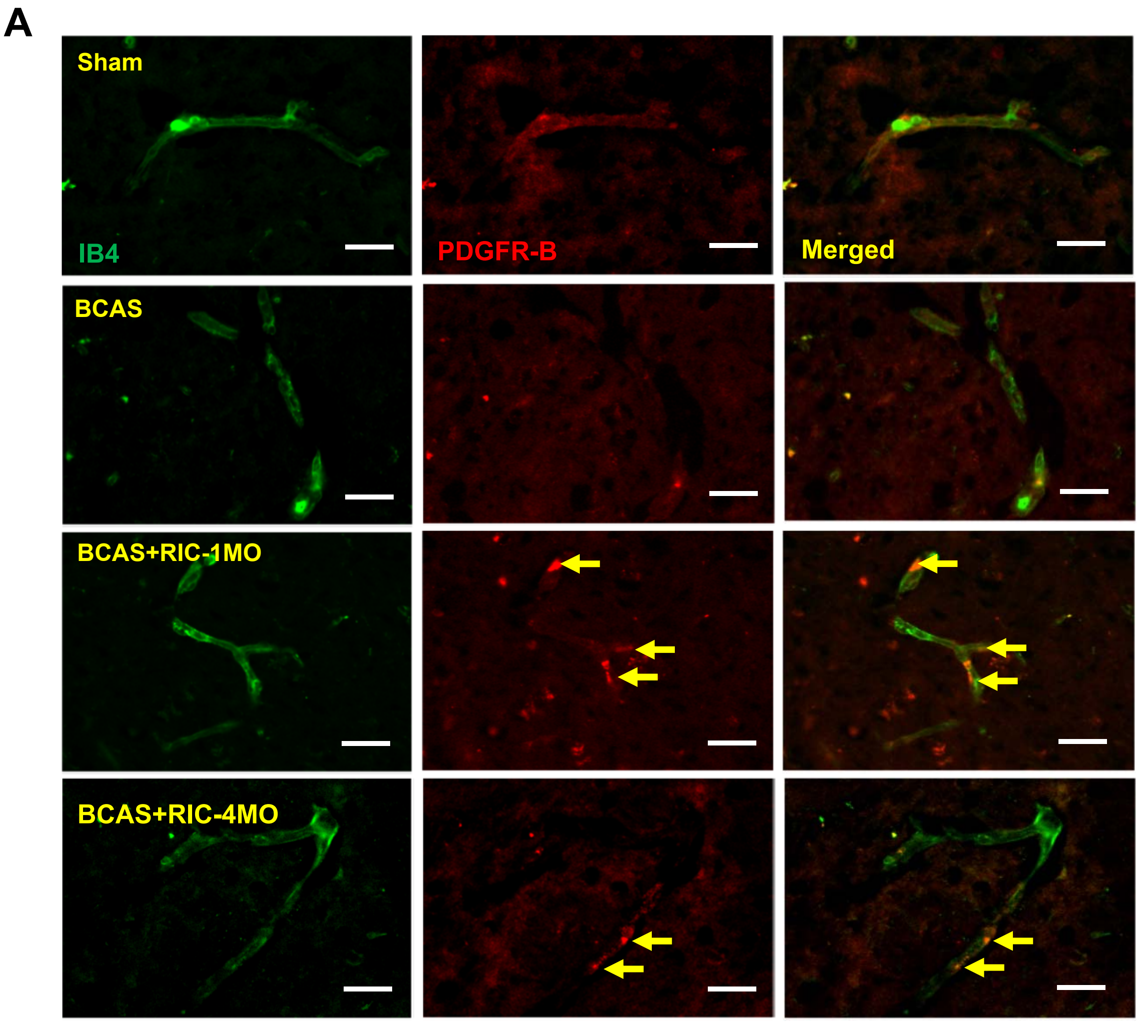

Supplemental Figure 6.

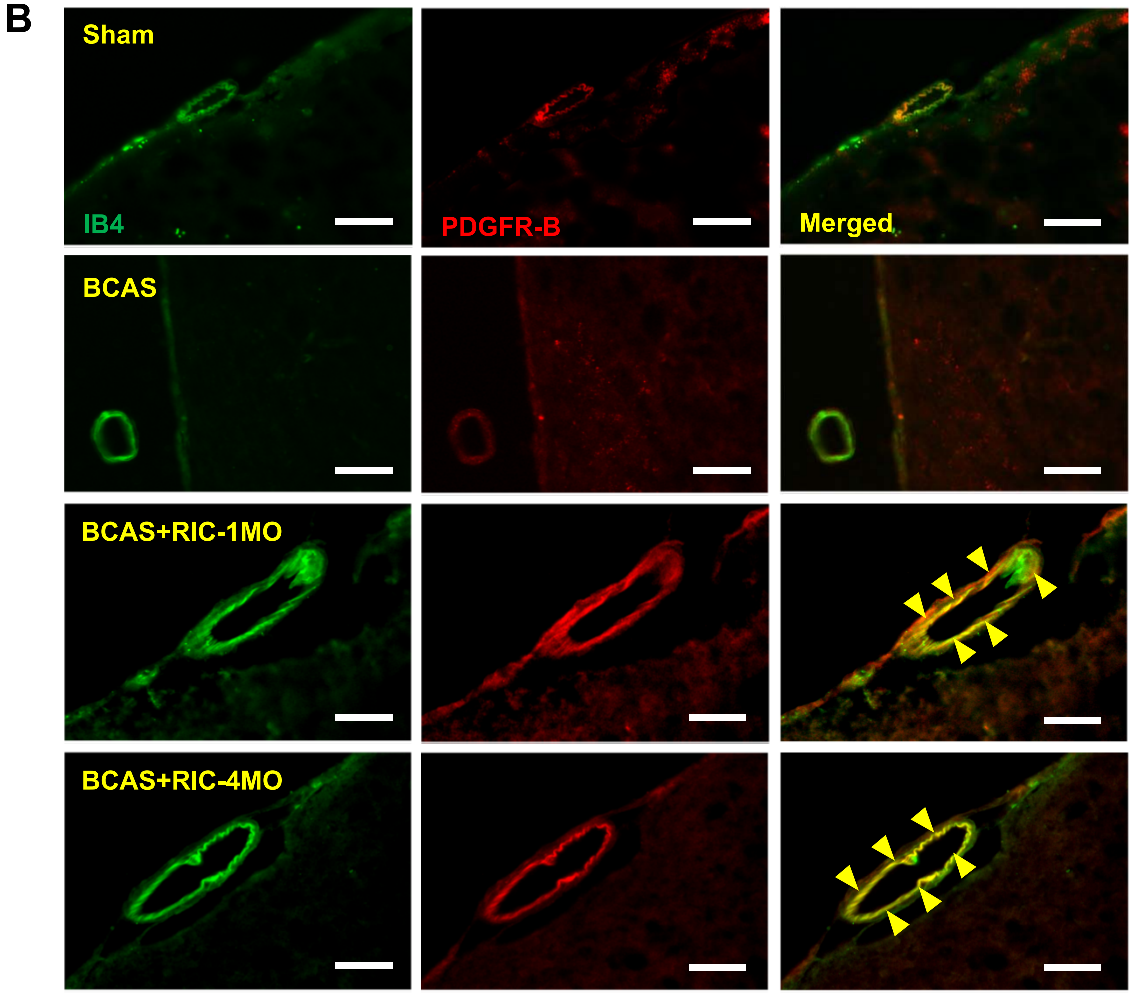

Supplemental Figure 6 (A-B). RIC can facilitate pericytes in BCAS mice via increased CBF. **A, B** representative photomicrographs of double immunofluorescence for Isolectin-B4 (green, marker for vessels) and PDGFR-B (red, a marker for pericytes) in the capillary/vessels of striatum (caudoputamen) (A) and boundary of cortical capillary/vessels (B) of each indicated group at 6 months with or without RIC therapy (scale Bar = 50 μm/40x).

Supplemental Figure 7.

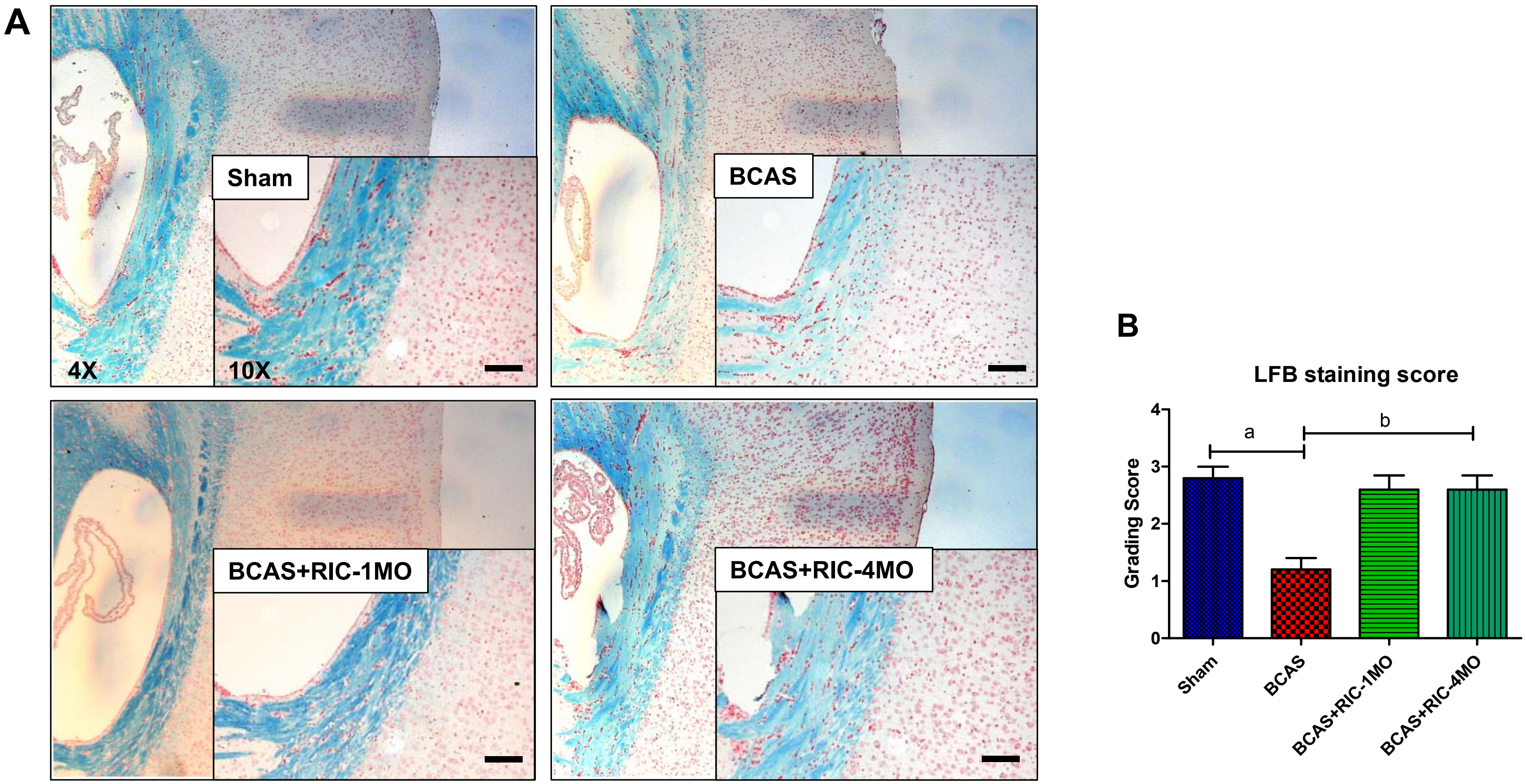

Supplemental Figure 7 (A-B). The intensity in Klüver-Barrera staining after BCAS was decreased than that after the sham operation while it was reversed by RIC therapy both in BCAS+RIC-1MO and BCAS+RIC-4MO groups, which have significant difference as compared to BCAS-Sham RIC groups. **A,** Representative images (4×) from cross sections of corpus callosum of the indicated groups of mice with the Luxol fast blue (LFB)/Klűver-Barrera staining and its higher magnification (inset, 10×, scale bar=20μm) showing significant degeneration of white matter after BCAS sham-RIC and its prevention by RIC therapy in both 1MO and 4 MO at 6 months. **B,** Histogram showing grading score of WM degeneration and their prevention by RIC. Values are indicated as mean ±SE. [a]p<0.0001 sham vs BCAS; [b]p<0.001 BCAS vs BCAS+RIC-1MO, BCAS+RIC-4MO.

Supplemental Figure 8.

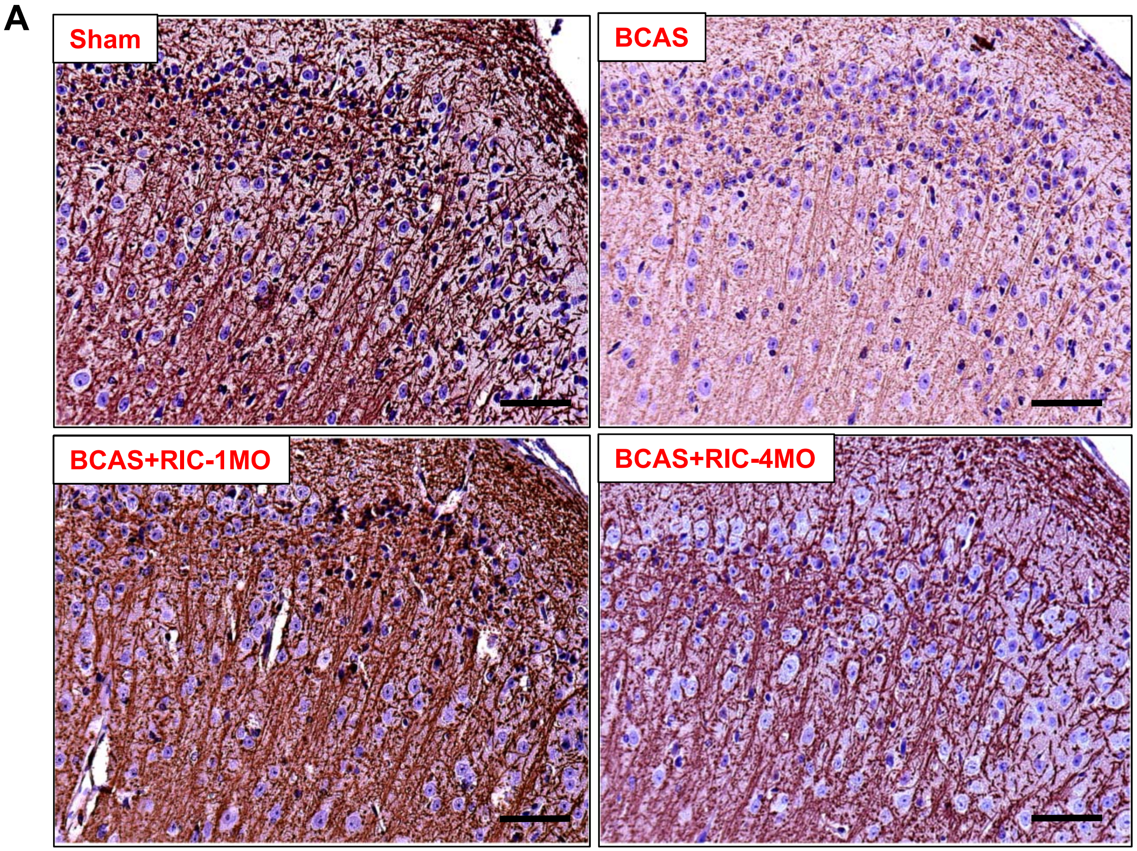

Supplemental Figure 8.

B

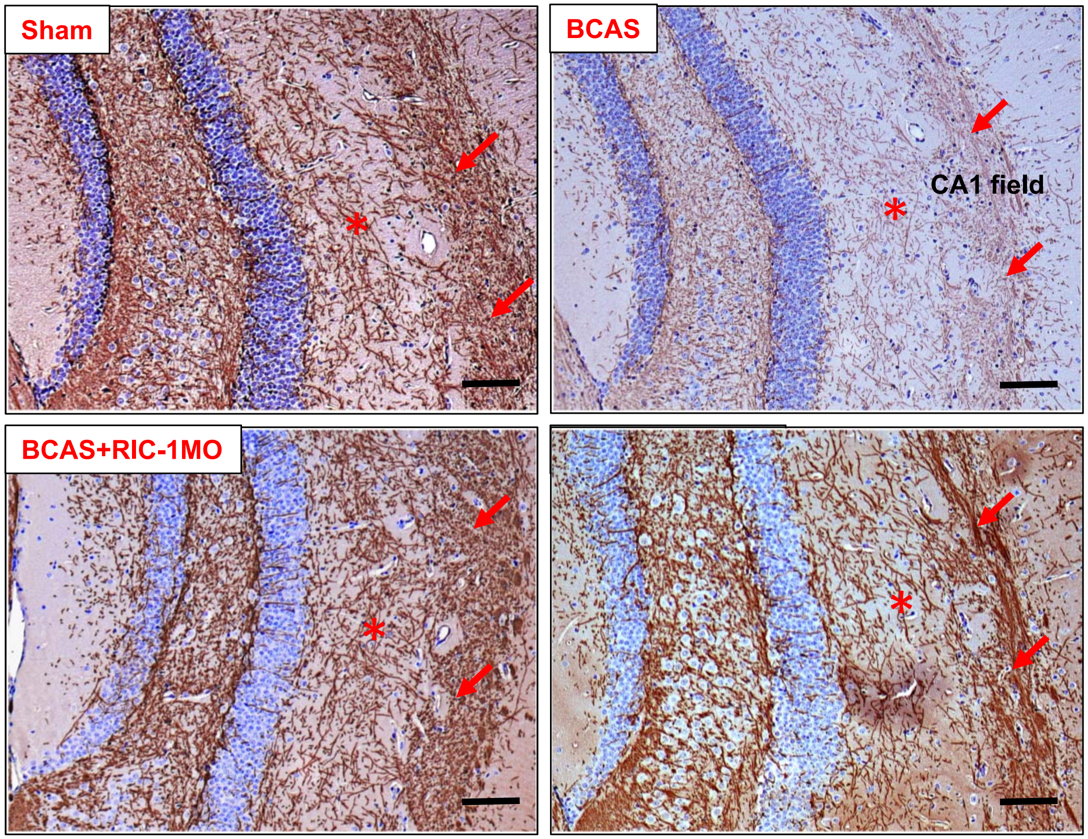

Supplemental Figure 8 (A-B). Myelin basic protein (MBP) involves in the process of myelination of nerves in the nervous system, and its expression often markedly declines during demyelination. We found the decrease of MBP staining was rescued by RIC therapy both in BCAS+RIC1-MO and BCAS+RIC4-MO groups as compared to BCSA (sham RIC) group. Together, these data indicate that implementation of C-RIC therapy can alleviate cognitive impairment and white matter damage after BCAS.

Loss in myelin basic protein (MBP) after sham and BCAS surgery, and its protection by RIC therapy. **A,** Representative images (20×, scale bar=50μm) from the cerebral cortical region showing significant loss in MBP after BCAS as detected by anti-MBP immunostaining (DAB, brown color). **B,** Representative images from the area showing CA1 field of hippocampus (10×, scale bar=20 μm) of the indicated groups of mice at 6 months after RIC therapy (1MO and 4MO) or Sham control.

Supplemental Figure 9.

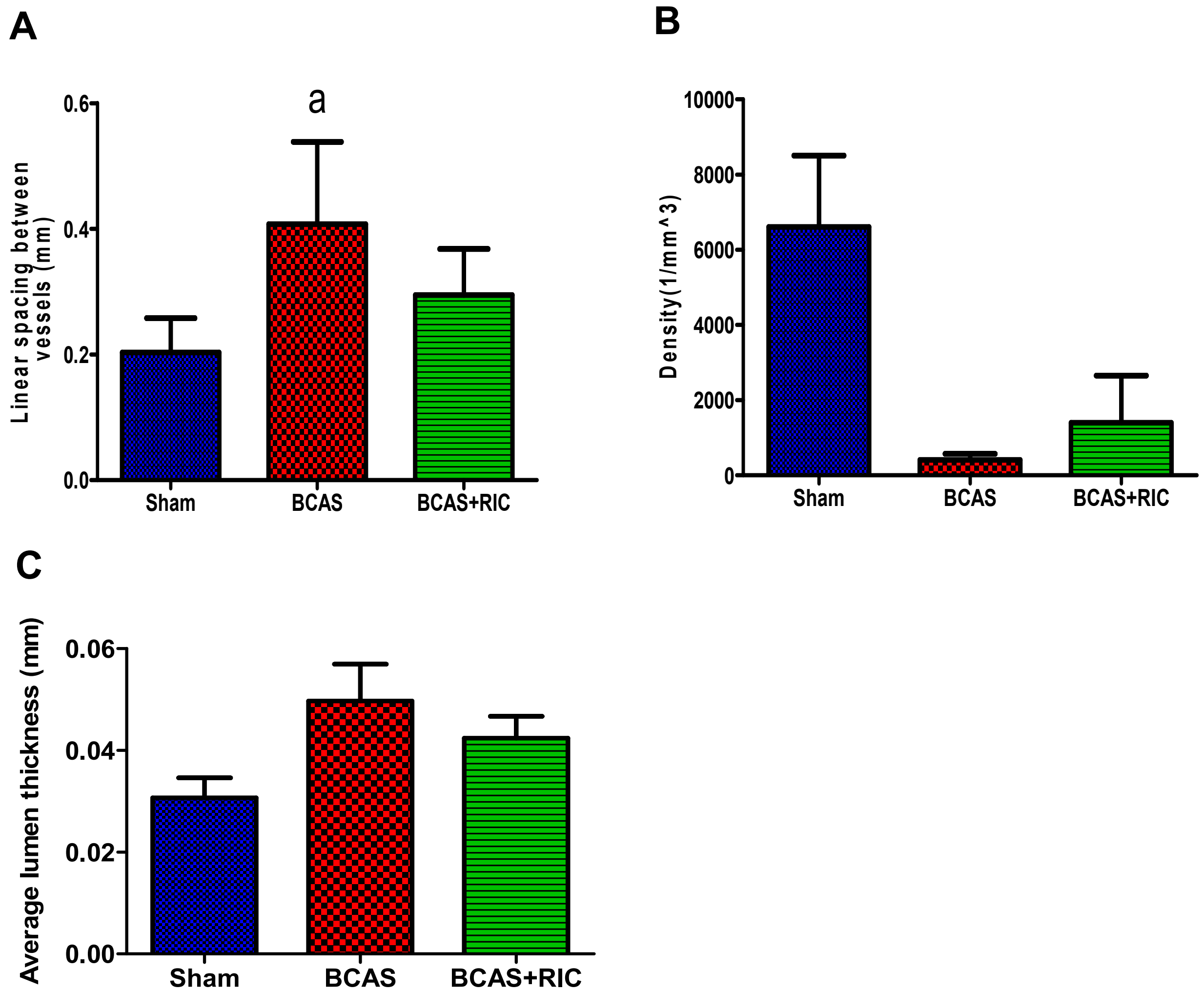

Supplemental Figure 9 (A-C). RIC improved the cerebrovascular angioarchitecture after 3 weeks treatment in young mice (10 weeks) after BCAS. A, Histogram showing linear space between the vessels, density (**B)** and lumen thickness **(C)** in all 3 groups after BCAS and RIC treatment**.** Values are indicated as mean ±SE. [a]$p<0.05$
